# Selective Reduction of THC’s Unwanted Effects through Serotonin Receptor Inhibition

**DOI:** 10.1371/journal.pbio.1002193

**Published:** 2015-07-09

**Authors:** Richard Robinson

**Affiliations:** Freelance Science Writer, Sherborn, Massachusetts, United States of America

## Abstract

How can we harness the beneficial effects of cannabis on pain without the harmful effects on memory? A new study shows that the harmful effects are specifically mediated by a partnership between cannabinoid and serotonin receptors, with pharmaceutical implications.

If it’s true that if you can remember the 1960s, then you weren’t there, it is likely in large part because of delta-9-tetrahydrocannabinol (THC), the major psychoactive ingredient in marijuana, one of whose effects is memory impairment. While recreational marijuana users may seek the full range of its effects, broad medical use of THC—including for pain, nausea, and anxiety—is hindered by them. In a new study, Xavier Viñals, Estefania Moreno, Peter McCormick, Rafael Maldonado, Patricia Robledo, and colleagues demonstrate that the cognitive effects of THC are triggered by a pathway separate from some of its other effects. That pathway involves both a cannabinoid receptor and a serotonin receptor, and when this pathway is blocked, THC can still exert several beneficial effects, including analgesia, while avoiding impairment of memory.

The recognition that there might be a connection between the two receptor systems is relatively recent. It is based on the fact that the two receptors are found in many of the same regions of the brain, that activation of the serotonin 5HT_2A_ receptor (5HT_2A_R) causes release of one type of endocannabinoid (a group of lipids that signal through cannabinoid receptors), and that loss of the cannabinoid CB_1_ receptor (CB_1_R) in mice disrupts serotonin-related activity in the prefrontal cortex.

This led the authors to explore whether the two receptors interacted at the molecular level. Using multiple assays, including a bioluminescent resonance energy transfer assay whose output signal reveals the proximity of two labeled molecules, they showed that 5HT_2A_R and CB_1_R form heteromers, binding with each other specifically and directly. When cells that expressed both receptors were treated with agonists for each, the authors found that downstream signaling was decreased by the combination of each agonist. Some of those downstream effects were synergistic for the two agonists, while others were not; full explanations of these varied effects will await further work.

The two receptors also displayed cross antagonism, in which an antagonist to CB_1_R prevented 5HT_2A_R signaling, and vice versa, further strengthening the case that the two receptors bind directly to each other. The authors convincingly argue that the cross antagonistic effect arises because each antagonist stabilizes the membrane-bound heterodimer in a conformation in which it cannot bind an intracellular G protein, required for transmitting the extracellular signal of THC or serotonin into the cellular interior.

The authors explored the behavioral consequences of this dual-receptor signaling using both 5HT_2A_R knockout mice and 5HT_2A_R antagonists in mice (Fig 1). In both cases, absence of 5HT_2A_R signaling reduced the amnesic effects of THC in a standard memory test; the anxiolytic effects, based on the amount of time the mice spent in an open aversive compartment; and the social facilitation effects, based on the amount of time the mice spent interacting with littermates. Reduction of all these effects could also be induced by administering a synthetic peptide that specifically prevented formation of the heterodimer, indicating its crucial role in mediating this subset of THC’s effects. But treatment to reduce 5HT_2A_R signaling did not change other effects of THC, including reduced movement, hypothermia, and analgesia, indicating that these effects are not mediated through the heterodimer.

**Fig 1:**
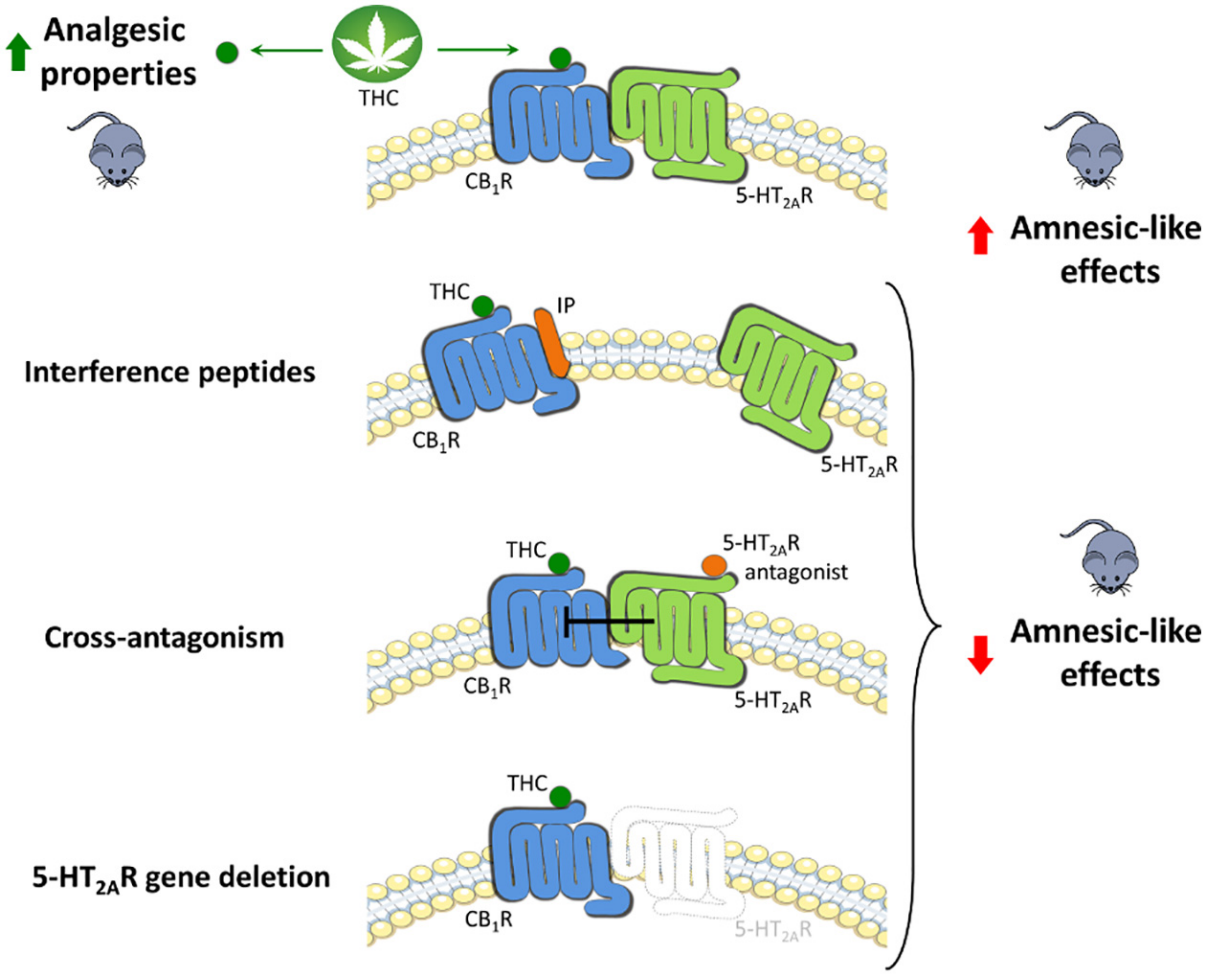
Can THC’s unwanted effects be selectively avoided? THC acts on CB_1_R to produce both detrimental and beneficial effects. We found that its undesirable amnesic properties are mediated through CB_1_-5-HT_2A_ heterodimers since disruption of this heterocomplex by interference peptides, pharmacological antagonism, or genetic deletion of 5-HT_2A_R leads to the specific blockade of memory deficits. *Image credit*: *Xavier Viñals and Estefanía Moreno*.

The results of this study are potentially highly important, in that they identify a way to reduce some of what are usually thought of as THC’s unwanted side effects when used for medicinal purposes while maintaining several important benefits, including pain relief. The widening acceptance of a role for THC in medicine may be accelerated by the option to reduce those side effects by selective pharmacological disruption or blocking of the heteromer.
